# BRCA1 founder mutations and ovarian cancer in Belarus

**DOI:** 10.1186/1897-4287-13-S1-A3

**Published:** 2015-09-09

**Authors:** A Savanevich, A Ashuryk, J Lubinski, J Gronwald

**Affiliations:** 1 Department of Obstetrics and Gynecology, Grodno State Medical University, Grodno, Belarus; 2 Department of Genetics and Pathology, Pomeranian Medical University, Szczecin, Poland

## 

In Belarus and other Slavic countries, founder mutations in the BRCA1 gene are responsible for a significant proportion of breast cancer cases. BRCA1 mutations also predispose to ovarian cancer. The aim of this study was to estimate the frequency of the three BRCA1 founder mutations in Belarus. The study group consisted of 134 consecutive, newly diagnosed cases of ovarian cancer after surgical treatment, unselected for age or family history. The control group constituted 853 blood samples from newborn children (417 girls and 436 boys). All patients were inhabitants of the western region of Belarus. DNA was extracted from peripheral blood lymphocytes for cases and from cord blood from afterbirth. Two BRCA1 mutations (4153delA and 5382insC) were studied using ASA-PCR and one mutation (C61G) was detected with RFLP-PCR. A mutation was identified in 9 of 853 newborn controls (1.1%). The 5382insC mutation was the most common (78%). A BRCA1 mutation was detected in 18 of the 134 (13.4%) unselected ovarian cancer cases. The median age of diagnosis of the 18 hereditary ovarian cancers was 54±10 years. (range 41–79 years). The frequency of a BRCA1 mutation in underlying population and hereditary proportion of ovarian cancer in Belarus is among the highest of any countries studied to date.

